# Enablers for resilience and pandemic preparedness in food supply chain

**DOI:** 10.1007/s12063-022-00272-w

**Published:** 2022-05-27

**Authors:** Mukesh Kumar, Rakesh D. Raut, Mahak Sharma, Vikas Kumar Choubey, Sanjoy Kumar Paul

**Affiliations:** 1 Department of Mechanical Engineering, National Institute of Technology Patna, Patna, 800005 India; 2 Department of Operations and Supply Chain Management, National Institute of Industrial Engineering (NITIE), Vihar Lake, NITIE, Powai, Maharashtra Mumbai, 400087 India; 3 Birla Institute of Management Technology, BIMTECH, Knowledge Park 2, NCR, Plot Number 5, BIMTECH Rd, Greater Noida, Uttar Pradesh, UP 201306 India; 4 Department of Mechanical Engineering, National Institute of Technology Patna, Patna, 800005 India; 5 UTS Business School, University of Technology Sydney, Sydney, Australia

**Keywords:** COVID-19 pandemic, Resilience, Preparedness, Food supply chain, Delphi-interpretive structural modeling, Fuzzy-DEMATEL

## Abstract

The recent COVID-19 pandemic has caused enormous disruptions to supply chain (SCs). Border restrictions forced countless businesses to close either permanently or temporarily. However, the food industry is an essential sector that needs to be operational during a pandemic. Although the food industry has proactively worked towards fulfilling human needs, the food supply chain (FSC) faced numerous challenges, forcing SC managers to rethink their business strategy to cater to consumer demands effectively. In a pandemic situation, manufacturing operations need to repurpose and adapt to produce different high-demand products. Resilience initiatives help fight disruption phases in an uncertain environment by building capacity to resist and recover to a better position. This study identifies 14 key enablers to develop a resilient FSC and reveals the most significant enablers in India. We used a hybrid Delphi-interpretive structural modeling (ISM) and Fuzzy decision-making trial and evaluation laboratory (Fuzzy-DEMATEL) methodology to achieve these goals. The Delphi technique identified essential enablers, while the ISM analyzed the interrelationship among enablers and level of importance in a hierarchical structural model. Finally, the Fuzzy-DEMATEL categorized the enablers into the cause-effect group. This study helps SC decision-makers recognize the enablers and the contextual and causal relationships to improve resilience initiatives. It also helps them repurpose their manufacturing operations and shift to other highly required and high-demand production.

## Introduction

A supply chain network (SCN) links customers, suppliers, and manufacturers (Carter and Rogers 2008). Several parties, such as suppliers, producers, warehouses, distributors, sellers, and consumers, are associated with the SCN (Luthra et al. 2018; Kumar et al. 2021). The customer is an essential part of the SCN because the demand for products is generated from them, and all other parties work towards fulfilling their demand (Carter and Rogers 2008). There is increasing modern SC complexity because products are distributed globally (Wang et al. 2020). Increasing complexity in the SC increases uncertainty, which multiplies manifold during unexpected events like disruptions (Jabbour et al. 2019). Interruptions in SC significantly affect the continuity of operation and economy (Ivanov and Dolgui 2020a; Namdar et al. 2018). Almost all SCs have faced many challenges such as uncertain demand, change in customer behavior, worker unavailability, lack of visibility, and the bankruptcy of supply chain partners because of the COVID-19 pandemic (Paul et al. 2021).

Manufacturing firms face an enormous challenge to keep their operations functional and run their businesses as previously. Due to the COVID-19 pandemic, manufacturing firms have the massive challenge of bridging the gap between demand and supply (Chowdhury et al. 2020). Due to a lack of pandemic preparedness, the recovery from massive disorder is slow in SC (Dubey et al. 2020). Thus, supply chain resilience plays a vital role in mitigating the effect of huge disruptions like a global pandemic. Therefore, it is important to identify the key enablers that enhance business performance and pandemic preparedness for the development of resilience practices in SC. Industries must be repurposed and shifted through pandemic preparedness to generate high-demand and vital products. Repurposing a manufacturing system is described as “structural and functional changes to a manufacturing system at a time when better opportunities and transition periods exist.” Several manufacturing systems have shifted and adapted their production facilities to generate different products in response to the pandemic. The wine business shifted to producing sanitizers, nitrogen filling plants produced oxygen, and textile manufacturers shifted to producing masks and gloves (Omezzine et al. 2021).

COVID-19 has had significant adverse effects on the economy, lead time, customer behavior, and productivity (Paul et al. 2021; Omar et al. 2021; Islam et al. 2021). To negate the impact of COVID-19 on SC, powerful recovery management strategies and resilient SC are required (Chowdhury et al. 2021). Lack of resistance in distribution and operation is the primary reason for SC failures during the recent COVID-19 pandemic (Paul and Chowdhury 2021). These SC interruptions are caused by worker unavailability, supplier failure, lockdown restrictions, boundary restrictions of states and countries, demand fluctuation, and changes in the customer behavior pattern (Kumar et al. 2020; OECD 2020). Manufacturers who expect zero or very little demand for their products during the pandemic have to adapt their manufacturing operations to other high-demand products (Omezzine et al. 2021). Supply chain operations must deliver products online through contactless delivery between the buyer and the seller. Resilient SCs have strong adaptive capabilities to restore themselves to the previous state (FAO 2020; Singh et al. 2020). It is worth noting that COVID-19 has delivered a vital lesson to SC that improper disruption risk management, generally on the supply side, severely disturbs all operations (Taleizadeh et al. 2021).

During the recent COVID-19 pandemic, several researchers addressed the necessity of mitigating the pandemic effect and restructuring the SC by adopting resilience practices. A resilient SC has an effective reactive, proactive, and recovery strategy. Resilience is a characteristic of any SC, helping to absorb adverse external turbulences and bring back the normal conditions (Hosseini et al. 2020). Resilience is equivalent to immune systems (the capacity to resist and restore) (Ivanov 2018). A healthy immune system in nature and people helps ensure strong performance. Similarly, SC performance is interrelated with resilience and sustainability. A low immune system has lower resiliency, thereby leading to poor performance. Low supply chain resilience (SCR) decreases profitability, mismatches demand and supply, and disrupts everyday operational strategies in ordering, production, inventory control, and distribution (Ivanov 2018). Therefore, resilience and an effective recovery strategy are vital for tackling pandemic-like crises (Chowdhury et al. 2021).

Food is one of the most essential and inevitable needs of human beings. Effective FSC positively contributes to a nation’s economy (Gardas et al. 2018). The recent COVID-19 pandemic affected the FSC’s ability to fulfill customer demand. The FSC faced several challenges in providing safe and secure food to consumers because consumer behavior and trust underwent total change during the pandemic. Indeed, the COVID-19 pandemic has sounded a warning bell for the FSC, indicating the urgency to improve food well-being, security, and flexibility, and put in place an effective recovery plan to fulfill consumers’ demand (Fan et al. 2021). During a pandemic, a flexible manufacturing firm repurposes its operations to create a new product or service (Poduval et al. 2021). The most prominent example of repurposing is the transformation of teaching and learning services, such as colleges and coaching, from offline to online classes. In the recent pandemic, FSCs have failed to execute resilience practice and operate business operations, while other sectors run their business by repurposing their manufacturing system. With the advantage of repurposing strategy in the pandemic period, business operation researchers are motivated and are contributing to the literature by analyzing resilient enablers for repurposing and pandemic preparedness in FSC.

The development of resilience practice in FSC results in the smooth distribution of food commodities even during mass disruptions (Ali et al. 2021; Sharma et al. 2022). Therefore, identifying and exploring the key resilient enablers that help in preparedness during the pandemic is vital to implementing resilience practice in FSC. Generally, enablers complement the drivers that enhance the development of resilience practice to fight several disruptions (like tsunamis, cyclones, pandemics, labor strikes, traffic blockages, etc.). In this study, several vital enablers have been discussed and analyzed in the context of the food supply chain. The COVID-19 pandemic has affected almost every supply chain, including FSC. Many food manufacturing firms were worst affected during the pandemic and faced considerable challenges in running their businesses because of worker unavailability, illness, and isolation. Food manufacturing firms from almost every country, including the USA, UK, Italy, Germany, and India, failed pronouncedly during the pandemic. On the other hand, while manufacturing firms with effective proactive and reactive strategies faced major challenges during the pandemic, they recovered well (Paul et al. 2021). It has also been observed that wine production plants modified their manufacturing systems to produce sanitizer, which produces profit during a pandemic (Malik et al. 2021; Poduval et al. 2021; Throup et al. 2021; Ozdemir et al. 2022). As a result, manufacturing system flexibility and resilience are crucial in a pandemic. To explain the significance of supply chain resilience, this study discusses the critical resilience enablers that help prepare for a pandemic or other disruptions, towards repurposing their production operations. This study attempts to answer the following questions.

- RQ1. What are the critical enablers for developing FSC resilience during a pandemic?
- RQ2. What are the contextual interrelationship and causal relations of the resilient enablers in the context of pandemic preparedness in FSC?
- RQ3. How do resilient enablers help prepare and repurpose the FSC during a pandemic?

Identifying FSC enablers is essential for developing resilience initiatives that can help firms prepare for pandemics (Ali et al. 2017). Enablers such as risk management culture, building analytical capabilities in FSC, creating agility, etc., are helpful in pre-disruption. They are also responsible for preventive capacity development (Lohmer et al. 2020). Enablers like ‘providing safety stock,’ ‘multi-sourcing,’ ‘collaboration among players,’ and ‘backup utility systems’ help build resistive capacity in the FSC during the disruption phase. Furthermore, several enablers, such as flexible suppliers, redundancy, and innovation in food traceability, are helpful during the post-disruption phase (Alemany et al. 2021). Thus, FSCs with recoverability and preventive and resistive capacities are likely to be sustained during the pandemic and post-pandemic periods. Some resilient enablers, including supply chain flexibility, market research, backup utility devices, and SC cooperation, can help repurpose a production system to quickly adapt its factory layout to produce another demanding product (Throup et al. 2021).

There is a dearth of research studying the interrelationship among the enablers that can help build new initiatives in FSC (Mangla et al. 2018). Several researchers have used different methods to establish contextual interrelationships among the enablers. Previous literature findings indicate that interpretive structural modeling (ISM) is a highly utilized methodology to model relationships among poorly connected variables (Balaji and Arshinder 2016; Gardas et al. 2019). Some researchers have used the DEMATEL methodology alongside the ISM technique to explore causal relationships with their intensities (Kumar and Dixit 2018; Yadav et al. 2020b). The Delphi methodology, based on experts’ inputs, has been utilized for selecting key variables (Paul et al. 2021). Interestingly, Naeini et al. (2019) applied the Delphi method with ISM and Grey DEMATEL to model competitive advantages in the food industry. This study aims to analyze enablers, explore contextual interrelationships, and classify them based on causal interaction (cause-and-effect) with respective intensities. A single MCDM technique cannot fulfill the objectives of this study. Therefore, an integrated Delphi-ISM-DEMATEL methodology is required.

During a pandemic, repurposing industrial systems and supply chain activities is an urgent requirement. Future manufacturing and supply chain operations need to be flexible and robust, changing their structure and operations in response to opportunities. Based on successful examples of repurposing during the COVID-19 pandemic, this study makes a novel contribution to the literature by analyzing the enablers for effective adoption of resilience practice in FSC for pandemic preparedness and repurposing their manufacturing system. The findings of this study will help practitioners and policymakers prepare for pandemics by developing resilience strategies. The enablers were selected by applying the Delphi technique. The interrelationship and hierarchical structure among the identified enablers were developed through the ISM and further clustered by MICMAC analysis. The interrelationship among the enablers helps to understand the enablers for adopting resilience. Furthermore, the Fuzzy-DEMATEL methodology was applied to discriminate the enablers into cause-and-effect groups and prominence and relational value. The results and findings of this study will be helpful for policymakers in addressing the development of resilience practices in FSC and ensuring pandemic preparedness.

The paper is organized as follows. Section 2 presents a literature review of previous relevant studies on resilience and pandemic preparedness in FSC. Section 3 describes the research methodology adopted in this study, while results are analyzed in Sect. 4. Discussions and implications for theory and practitioners have been provided in Sect. 5. In Sect. 6, the study is concluded with limitations and future research directions.

## Literature review

In this section, previous literature contributions are discussed. First, we discuss the various risks and challenges of the FSC. Second, how industries tackle those challenges and risks associated with the FSC are discussed, followed by a discussion on resilience in the FSC. The distinct methodologies used to explore the enablers that help build resilience in SC management are also discussed in detail.

### Food supply chain and disruptions

Manufacturing firms have faced severe disruption in their operations due to this pandemic. Blos and Wee (2020) reported that manufacturing firms were affected significantly by approximately 1400 epidemics in the last ten years. However, food is an essential commodity, and operational failure in this sector can prove calamitous. The operational failure reported in FSC during a pandemic is primarily because of the considerable variation in volume to sales ratio, demand fluctuation, distribution failure due to boundary restrictions, price controls, logistics failures, and lack of IT integrated systems (Leat and Revoredo-Giha 2013; Singh et al. 2020; Chowdhury et al. 2021; Taleizadeh et al. 2021). Artificial intelligence may help FSC lower waste by adopting circular economy practice and generating economy (Agrawal et al. 2021). FSC has significant challenges in resisting the competitive and uncertain business environment. The food sector's main challenges during the COVID-19 pandemic include meeting customer expectations and safely distributing nutritious and hygienic food (Gholami-Zanjani et al. 2021b). Evidence shows that customer behavior regarding food can change significantly during a pandemic or epidemic situation (Singh et al. 2020; Guthrie et al. 2021; Omar et al. 2021). There has been a significant change in the pandemic demand pattern, from hotels and restaurants to supermarkets (OECD 2020). Therefore, during disruptions like a pandemic, forecasting accuracy and regular market survey for customer behavior and competitor strategy are significant to help cope with the demand uncertainty (Gružauskas et al. 2019). During the pandemic, the demand for various safety and hygiene items, such as surgical masks, face masks, ventilators, oxygen concentrators, and testing kits, has increased dramatically (Poduval et al. 2021). In response to the shift in demand, several manufacturing plants have shifted their operations to produce those high-demand products (Throup et al. 2021). Poduval et al. (2021) identified challenges that manufacturing companies encounter while transitioning from one production operation to another in a short period. The biggest challenges to repurposing industrial activities have been identified as strategic, technological, cultural, and innovation barriers.

Disruption and risk are widespread and unavoidable concerns for businesses, and while the COVID-19 pandemic has exposed the FSC's real problem, it has also given opportunities for the future (FAO 2020). Disruptions occur in SCN because of natural events such as earthquakes, floods, and pandemics, and also by man-made events such as labor strikes and traffic congestions, all of which significantly disturb functional operations of FSCs (Yang and Xu 2015; Umar et al. 2017; Chowdhury et al. 2021). The impacts of natural disruptions are more uncertain and severe as compared to man-made events (Barman et al. 2021). During a pandemic, there is enough food commodity available in stock, but logistic failures and lack of traceability prevent consumers from getting sufficient food (Barman et al. 2021).

Several researchers have studied various strategies to tackle the disruptive pandemic event optimally. Barman et al. (2021) and Paul and Chowdhury (2020) have suggested recovery strategies like maintaining employees’ safety and health, changing working conditions, and emergency sourcing and collaboration, among others, to tackle the impacts of an epidemic. Apart from recovery strategies, several authors suggested developing resilience practices in the SC for handling pandemic situations (Singh et al. 2021; Chowdhury et al. 2020; Gholami-Zanjani et al. 2021b).

### Resilient food supply chain for repurposing and pandemic preparedness

Resilient supply chains have various capabilities such as resistive capacity, adaptive capacity, restorative capacity or recoverability, and risk management ability (Gholami-Zanjani et al. 2021b; Singh et al. 2020). The SC's adaptive capability helps prepare for an unforeseen and dynamic environment, respond to interruption, and recuperate from the disruption by continuing operations with the required degree of connectedness and structure control, which is essential to survive massive disruptions (Taleizadeh et al. 2021). A resilient SC must possess these abilities to absorb external and internal shocks, adapt even in the uncertain and dynamic business environment, and resist disruptions in reactive and proactive ways (Hosseini et al. 2019; Zavala-Alcívar et al. 2020). Generally, two strategies have been used in SCs to build resilience: proactive and reactive (Vroegindewey and Hodbod 2018; Ivanov and Dolgui 2020b; Qingbin et al. 2020). The proactive strategy helps prepare before the disruption, while the reactive approach works after disruption to ensure restoration to the initial state (Gunessee and Subramanian 2020).

Resilience in the SC also increases robustness by improving leanness and flexibility (Sehnem et al. 2019). It is the function of agility. In contrast, agility is related to flexibility and leanness, leading to resilience by empowering businesses to predict and respond to the interruptions evolving from the demand side of SC (Purvis et al. 2016). Xu et al. (2020b) provided a set of resilience-based endurance strategies to support organizations to train themselves better and minimize hazards by improving visibility and sensitivity to interruptions in a complex global supply chain. Gholami-Zanjani et al. (2021a) designed a resilient food SCN under epidemic disruption by focusing on product perishability and discount price based on product age through dynamic modeling and Monte Carlo simulation. Ivanov and Dolgui (2020b) developed the influence of viability in intertwined SCN by extending the concept of resilience to viability. They stated that the digital supply chain, including big data and blockchain, also helps develop resilience practice and extend viability in SC. Qingbin et al. (2020) analyzed COVID-19 impact on Chinese dairy SC and concluded that the resilience practice keeps firms safe from disruptions and provides strength during the recovery process. The previous studies on resilience practice within the supply chain are presented in Table 1.

**Table 1:** Previous studies on supply chain resilience

Author	Area/theme	Methodologies	Objectives	Findings
Brandon-Jones et al. 2014	Supply chain	Confirmatory factor analysis and multiple regression analysis	To explore the relationship among information sharing and connectivity, visibility, and performance	The supply chain resilience and robustness are increased by SC connectivity and information sharing lead visibility
Ali et al. 2017	Perishable food	Content analysis	To develop a framework to build resilience in Australia SMEs	The level of resilience varies within SMEs based on their size and nodes in the SC
Hecht et al. 2019	FSC	Semi-structured interview and phronetic iterative approach	How organizational level factors of food system resilience may impact disaster response	Factors like formal emergency planning, food suppliers, staff training, and the redundancy of the food system provide resilience
Dubey et al. 2020	Humanitarian Supply chain (HSC)	Structural equation modeling	To explore how blockchain enhances swift trust, collaboration, and resilience within HSC	Blockchain can improve SC resiliency and swift trust among the partners engaged in disaster relief operations
Hosseini et al. 2020	Supply chain	Bayesian network modeling	Measurement of supply chain resilience	Supply chain resilience measurement and conceptualization framework have been developed using the Bayesian network approach to balance risk mitigation and recovery capabilities
Thilmany et al. 2021	Local and regional FSC	Survey-based conceptual study	To examine the role of local and regional FSC during COVID-19	Local and regional FSC firms appear to be agile and well-connected to SC partners, allowing them to develop rapidly and with a focused strategy
Fan et al. 2021	Food supply chain	Survey-based conceptual study	Analyze the impacts of COVID-19 on Asian FSC	The global FSC severely impacted Covid-19 as compared to the local food system. Through resilience practice, FSC can mitigate the future pandemic
Guthrie et al. 2021	E-commerce	Pattern matching process	To study consumer behavior patterns before and after Covid	In the Covid pandemic, consumers shifted from retail business to e-commerce platform
Ivanov and Dolgui 2021	Supply chain	Modeling and data-driven decision modeling approach	To develop digital SC twin for risk and resilience	SC urgently needs digital SC twins to ensure viability, risk management, and mapping of the SC network
Taleizadeh et al. 2021	Inventory control	Optimization and uncertainty modeling	To examine the role of inventory management on supply-side disruption	Suggested that keeping a suitable amount of backorders is helpful during supply-side disruption

**Table 2:** Advantage of proposed methodology over other methods of the same group

Attributes	ISM-MICMAC	DEMATEL	ISM-DEMATEL	DELPHI-ISM-DEMATEL	Structural equation modeling
Input	Pairwise relationship for each factor	Pairwise importance value for each factor	Pairwise relationship, an importance value for each factor	Survey data for each factor, pairwise relationship, an importance value for each factor	Survey data for each factor
Advantage	Only contextual interrelationships among the factors were studied	It discusses the relationship intensity and the cause-and-effect category	It provides contextual interrelationships; their importance and intensity are also categorized into cause and effect	It provides the same results as ISM-DEMATEL, but in addition to Delphi, it analyzes only the relevant and critical factors	SEM tests the relationship among the factors with their significance level
Disadvantage	The level of intensity for relationship and significance level is missing	It does not provide a hierarchical structure and the significance level	The significance level for the relationship is not available	The significance level for the relationship is not available	Causal interaction and hierarchical structure are missing

**Table 3:** Demographic information of the experts with satisfactory replies

Designation	Industry	Qualification			Experience		Number of expert(s)	M/F
		Graduate	Post Graduate	Ph.D	5–10 years	> 10 years		
Executive manager	Dairy processing	1	4	0	2	3	5	3/2
Production manager	Beverage	4	5	0	3	6	9	6/3
Supply Chain Executive	Food processing	10	4	1	7	8	15	10/5
Marketing manager	Food processing	8	10	1	8	11	19	13/6
Professor	Food Supply Chain	0	1	6	0	7	7	4/3
Assistant professor	Food Supply Chain	0	2	3	1	4	5	3/2
**Total**		23 + 26 + 11 = 60			21 + 39 = 60		60	39/21

**Table 4:** Selection of enablers for development of resilience in food supply chain

Number	Enabler	Mean	Median	Standard Deviation	Accept/ Reject
1	Innovation in food traceability	4.8000	5.0000	0.44721	Accept
2	Use of local and regional food systems	4.8000	5.0000	0.44721	Accept
3	Allow suitable/optimum backordering	2.6000	3.0000	0.54772	Reject
4	Collaboration among supply chain players through information sharing, confidence, and trust-building	4.4000	4.0000	0.54772	Accept
5	Responsiveness to customer need and competitor strategy	4.4000	4.0000	0.54772	Accept
6	Multi-sourcing	4.4000	4.0000	0.54772	Accept
7	Market research of customer behavior pattern	4.6000	5.0000	0.54772	Accept
8	Build analytic capability to identify risk and uncertainty and respond quickly	4.6000	5.0000	0.54772	Accept
9	Providing safety stock	4.6000	5.0000	0.54772	Accept
10	Business certification	2.8000	3.0000	0.83666	Reject
11	Training and development	3.8000	4.0000	0.83666	Reject
12	Quality management	3.8000	4.0000	0.83666	Reject
13	Application of AI and Big data	3.8000	4.0000	0.83666	Reject
14	Building risk management culture leadership and risk innovation	4.2000	4.0000	0.83666	Accept
15	Supplier alert management	4.2000	4.0000	0.83666	Accept
16	Creating flexibility within the supply chain by using the backup utility system and flexible supplier	4.0000	4.0000	1.00000	Accept
17	Intertwined supply network	3.8000	4.0000	1.09545	Reject
18	Creating agility through velocity and visibility by incorporating new technologies	4.2000	5.0000	1.09545	Accept
19	Downstream decoupling point	4.0000	4.0000	1.22474	Accept
20	Creating redundancy within supply chain infrastructure and inventory	4.0000	4.0000	1.22474	Accept
21	Supply chain globalization	3.8000	4.0000	1.30384	Reject
22	Promising food security	3.8000	4.0000	1.30384	Reject

>Respondent, please give your opinion for the enablers on a 1–5 scale. How are the enablers important for building resilience in FSC for pandemic’s preparedness?

**Table 5:** Identified resilient enablers for pandemic preparedness

Identified Resilient Enablers for pandemic preparedness	Description	Source
Innovation in food traceability (E1)	Implementing traceability is a proactive approach to increase transparency within the supply system. In a pandemic situation, food traceability facilitates real-time inventory tracking and efficient logistics management, effectively controlling inventory and improving customer service and quality	Epelbaum and Martinez (2014); DiMase et al. (2016); Saberi et al. (2019); Ali et al. (2017)
Use of local and regional food systems (E2)	In the current pandemic, local and regional food systems have played an important role in building the resilience of FSC. Local or regional food systems help build rapid recovery capacity and improve resilience	Béné (2020); FAO (2020); Thilmany et al*.* (2021); (Hobbs (2020); Dirsehan and Cankat (2021)
Market research of customer behavior pattern (E3)	In the current pandemic situation, customer behavior has undergone significant changes, leading to large fluctuations in demand. Therefore, the market research to study customer behavior patterns acts as a proactive and recovery element while building resilience in the food industry	FAO (2020); Mishra and Shekhar (2013); Dirsehan and Cankat (2021)
Build analytic capability to identify risk and uncertainty and respond quickly (E4)	In the modern era, supply chains operate on a global scale, increasing risks and uncertainties. Therefore, to resolve SC uncertainty and ensure a quick response, it is necessary to establish analytical capabilities in the SC to perform risk assessment and mitigation	Meuwissen et al. (2019); Vroegindewey and Hodbod (2018); Raut et al. (2021)
Providing safety stock (E5)	Manufacturing operations based on just-in-time production have been severely affected by the pandemic because it does not allow for safety stock, thus impairing customer service. Therefore, enabling safety stocks in inventory helps build supply chain resilience by resisting demand uncertainty and supply disruption	FAO (2020); Taleizadeh et al. (2021); Stone and Rahimifard (2018); Hosseini et al. (2019); Paul et al. (2021)
Collaboration among supply chain players through information sharing, confidence, and trust-building (E6)	To withstand a disruption like the COVID-19 pandemic, sharing information between SC players and trust-building between them is important. Therefore, stakeholders have an important role in restoring their original financial and social situation in an emergency	Singh et al. (2018); FAO (2020); Alemany et al. (2021); Paul et al. (2021)
Responsiveness to customer needs and competitor strategy (E7)	The responsiveness to customer requirements helps to fight against demand fluctuation and build reactive capacity in the SC. Reactive capacity within the SC is essential for developing resilience and fighting against the pandemic situation for quick response	Ivanov (2018); Zavala-Alcívar et al. (2020); Ali et al. (2017)
Multi-sourcing (E8)	Through the multi-sourcing concept, the company uses multiple suppliers to provide the same materials or maintain a backup supplier to help during disruptions. The multi-sourcing strategy allows the firms to quickly switch to alternate suppliers when the main supplier is interrupted	Hosseini et al. (2019); Ivanov (2018); Paul et al. (2021)
Creating agility through velocity and visibility by incorporating new technologies (E9)	The SC's velocity and visibility are increased by introducing modern features like drones, smart sensors, traceability that help build agility in the SC and provide secure service to the customer	Yadav et al. (2020a); Zhu and Krikke (2020)
Building risk management culture, leadership, and risk innovation (E10)	In modern times, there are many risks in FSC due to its complexity. Thus, risk management principles act as a proactive element, effectively helping to reduce risks and avoid upcoming disruptions. Therefore, innovations in risk management and mitigation strategies are needed	Zhu and Krikke (2020); Leat and Revoredo-Giha (2013)
Supplier alert management (E11)	During the Covid19 pandemic, suppliers did not provide raw materials to companies. There are multiple risks on the part of the supplier, such as logistics failures, unavailability of materials, strikes, etc. Therefore, supplier alert management helps to overcome supplier-side disruption and increase resilience	Zhu and Krikke (2020); Taleizadeh et al. (2021); Stone and Rahimifard (2018)
Creating flexibility within the supply chain by using a backup utility system and flexible supplier (E12)	Resilience in FSC is improved through flexibility. Hence, flexible supplier and backup utility systems in the SC improve sustainability and resilience. Flexibility in the supply chain may increase with the addition of backup utilities that provide rigidity to mitigate operational risks under pandemics	Johnson et al. (2013); Zavala-Alcívar et al. (2020); Hosseini et al. (2020); Xu et al. (2020b); Paul et al. (2021)
Downstream decoupling point (E13)	The decoupling point in the downstream supply chain refers to the multiple storage facilities and control systems between the manufacturer and the market. Decoupling points enable the supply chain to reduce the risk of demand, logistics failures, and the establishment of self-sufficiency. Subsequent decoupling points will help increase resilience and sustainability to restart FSC's supply and delivery system	Zhu and Krikke (2020); Gold et al. (2017)
Creating redundancy within supply chain infrastructure and inventory (E14)	Redundancy establishes the mainstream of proactive and recovery strategies for interruption risks, while redundancy in supply chain infrastructure and inventory helps cope with uncertainty in supply and demand, thereby increasing resilience	Namdar et al. (2018); Tukamuhabwa et al. (2015); Hecht et al. (2019); Hosseini et al. (2019)

**Table 6:** SSIM for resilient food supply chain enablers

Resilient food supply chain enablers	E14	E13	E12	E11	E10	E9	E8	E7	E6	E5	E4	E3	E2	E1
E1	V	V	V	V	O	V	O	V	V	O	V	O	O	X
E2	V	O	V	V	O	V	V	V	O	O	V	A	X	
E3	V	O	O	O	O	O	O	V	O	O	V	X		
E4	A	O	A	A	X	A	O	V	A	O	X			
E5	V	A	V	A	O	O	A	V	O	X				
E6	O	V	V	O	V	A	O	V	X					
E7	A	A	A	A	O	O	A	X						
E8	V	O	V	O	O	V	X							
E9	A	O	A	A	V	X								
E10	O	O	V	A	X									
E11	O	O	V	X										
E12	A	A	X											
E13	V	X												
E14	X

**Table 7:** Level of importance and corresponding reachability and antecedent set for the enablers

	**Reachability set**	**Antecedent set**	**Level**
E1	1	1	9^th^
E2	2	2,3	9^th^
E3	3	3	10^th^
E4	4,10,12	1,2,3,4,5,6,8,9,10,11,12,13,14	2^nd^
E5	5	1,2,5,6,8,11,13	5^th^
E6	6	1,2,6,8,11	7^th^
E7	7	1,2,3,4,5,6,7,8,9,10,11,12,13,14	1^st^
E8	8	2,3,8	8^th^
E9	6,9,13	1,2,3,5,6,8,9,11,13,14	3^rd^
E10	4,9,10,12	1,2,3,4,6,8,9,10,11,12,14	2^nd^
E11	11	1,2,3,11	8^th^
E12	4,6,9,10,12	1,2,3,5,6,8,9,11,12,13,14	3^rd^
E13	13	1,6,13	6^th^
E14	6,14	1,2,3,5,6,8,11,13,14	4^th^

**Table 8:** Initial relation matrix

	E1	E2	E3	E4	E5	E6	E7	E8	E9	E10	E11	E12	E13	E14
E1	0	0	0	3	2	3	4	0	4	4	4	4	4	4
E2	0	0	0	3	4	3	3	4	4	2	2	4	0	4
E3	0	3	0	4	0	0	4	1	2	4	1	4	0	2
E4	0	0	0	0	0	0	4	0	0	4	0	4	0	0
E5	0	0	0	2	0	0	3	0	2	0	0	3	0	4
E6	0	0	0	1	1	0	2	0	1	2	0	3	3	2
E7	0	0	0	0	0	0	0	0	0	0	0	0	0	0
E8	0	0	0	2	4	3	3	0	2	2	0	4	0	4
E9	0	0	0	4	0	3	4	0	0	2	0	4	2	0
E10	0	0	0	4	0	0	3	0	3	0	0	4	0	0
E11	0	0	0	4	3	2	4	0	3	3	0	4	0	4
E12	0	0	0	3	0	1	4	0	4	2	0	0	0	0
E13	0	0	0	3	3	0	4	0	4	0	0	4	0	4
E14	0	0	0	3	0	2	3	0	2	1	0	4	0	0

**Table 9:** Total relation matrix

	E1	E2	E3	E4	E5	E6	E7	E8	E9	E10	E11	E12	E13	E14	**R**
E1	0.029	0.031	0.029	0.167	0.085	0.118	0.202	0.032	0.166	0.150	0.111	0.193	0.123	0.137	1.571
E2	0.029	0.031	0.029	0.158	0.124	0.121	0.179	0.109	0.158	0.111	0.071	0.173	0.045	0.138	1.475
E3	0.025	0.089	0.025	0.159	0.040	0.051	0.175	0.048	0.110	0.140	0.047	0.166	0.035	0.085	1.197
E4	0.019	0.020	0.019	0.051	0.025	0.030	0.135	0.021	0.047	0.114	0.022	0.127	0.024	0.029	0.683
E5	0.021	0.022	0.021	0.096	0.028	0.038	0.130	0.023	0.088	0.046	0.024	0.123	0.029	0.110	0.799
E6	0.022	0.024	0.022	0.080	0.049	0.038	0.114	0.024	0.074	0.085	0.025	0.129	0.092	0.079	0.858
E7	0.015	0.016	0.015	0.030	0.020	0.022	0.034	0.017	0.028	0.025	0.017	0.032	0.019	0.023	0.313
E8	0.025	0.027	0.025	0.116	0.112	0.108	0.154	0.027	0.105	0.097	0.028	0.162	0.038	0.125	1.150
E9	0.022	0.024	0.022	0.139	0.034	0.098	0.155	0.025	0.058	0.091	0.026	0.147	0.072	0.040	0.953
E10	0.020	0.022	0.020	0.132	0.027	0.036	0.130	0.022	0.108	0.048	0.023	0.136	0.029	0.031	0.785
E11	0.020	0.022	0.020	0.135	0.043	0.044	0.141	0.022	0.110	0.064	0.023	0.140	0.029	0.056	0.871
E12	0.020	0.022	0.020	0.116	0.028	0.051	0.142	0.022	0.121	0.084	0.023	0.062	0.030	0.032	0.776
E13	0.023	0.025	0.023	0.133	0.093	0.045	0.164	0.026	0.136	0.056	0.027	0.155	0.034	0.119	1.059
E14	0.021	0.023	0.021	0.119	0.029	0.075	0.132	0.023	0.089	0.065	0.024	0.139	0.032	0.035	0.828
**C**	0.313	0.397	0.313	1.632	0.737	0.877	1.986	0.441	1.398	1.176	0.492	1.885	0.632	1.039

**Table 10:** Prominence, relational vector, and rank of enablers

Enablers	R + C	R–C	Cause-effect	Influential Rank	Prominence rank	Relational rank
E1	1.884263	1.258403	Cause	5	6	1
E2	1.872141	1.078585	Cause	6	7	2
E3	1.509725	0.883865	Cause	9	13	3
E4	2.315229	-0.94908	Effect	3	3	12
E5	1.535641	0.062057	Cause	13	12	7
E6	1.734259	-0.019	Effect	12	9	8
E7	2.298486	-1.67263	Effect	2	4	14
E8	1.591204	0.708297	Cause	11	11	4
E9	2.351512	-0.44534	Effect	4	2	11
E10	1.961666	-0.3907	Effect	7	5	10
E11	1.362963	0.379129	Cause	14	14	6
E12	2.660317	-1.10898	Effect	1	1	13
E13	1.691068	0.426112	Cause	10	10	5
E14	1.867608	-0.21071	Effect	8	8	9

Chowdhury et al. (2021) developed a three-dimensional (preparedness, response, and recovery) resilience strategy for managing the impact of COVID-19 on SCs. Other researchers have identified several resilience strategies for pandemic preparedness, such as distributed manufacturing systems (Shokrani et al. 2020), providing backup suppliers (Ivanov and Das 2020), improving viability (Gunessee and Subramanian 2020) near sourcing (Zhu and Krikke 2020), and supply chain collaboration (Gunessee and Subramanian 2020). Allowing backordering in the supply chain helps build adaptability and resilience during periods of demand fluctuations (Taleizadeh et al. 2021). It has been observed that all manufacturing enterprises are under government pressure to employ renewable energy rather than non-renewable energy (Wilson et al. 2021). As a result of the energy shift, manufacturing activities are repurposing (Omezzine et al. 2021). Flexibility, cooperation, establishing agility via velocity, and responsiveness are some of the most robust enablers for building resilience and assisting manufacturing and supply chain processes in shifting swiftly (Throup et al. 2021). Because increased unpredictability leads to changes in demand for products, manufacturing systems that are flexible and agile are in high demand (Omezzine et al. 2021). However, identifying and modeling key enablers are critical for developing new initiatives in firms (Ali et al. 2017). At the same time, there is a dearth of studies that have comprehensively explored enablers in FSC in the context of pandemics or massive disruption events.

### Tools, techniques, and critical enablers used in previous studies

Researchers have extensively studied enablers while developing new initiatives for sustainable FSC (Mangla et al. 2018; Hussain and Malik 2020; Kumar and Choubey 2021), resilient SC (Soni et al. 2014), and green SC management in FSC (Malviya and Kant 2017). Previous research works have also analyzed critical success factors (CSF) for flexible green SC and sustainability initiatives in SC (Luthra et al. 2018) in different sectors such as automobile (Gopal and Thakkar 2016) and reusable plastic packaging (Gardas et al. 2019). Previous studies on the modeling of enablers are shown in Appendix Table 14.

Different enablers used by researchers in developing resilience practice are: appropriate supplier selection (Ali et al. 2017), building logistic capability (Béné 2020; Leite and Kumar 2021), cooperation (Scholten and Schilder 2015), creating public–private collaboration (Tukamuhabwa et al. 2015; Gunessee and Subramanian 2020), risk management culture (Taleizadeh et al. 2021), increasing visibility (Ivanov and Dolgui 2020b), and implementing traceability (Epelbaum and Martinez 2014; Tukamuhabwa et al. 2015; Queiroz et al. 2020). These enablers are used to develop proactive strategies during contingency planning (Vroegindewey and Hodbod 2018), create redundancy (Zavala-Alcívar et al. 2020) and SC collaboration (Scholten and Schilder 2015). However, Naz et al. (2021) stated that artificial intelligence is the enabler of SC resilience post-COVID-19 pandemic to support preparedness and help in risk management.

ISM has been extensively used in modeling the multi-criteria problem in FSCs (Sagheer et al. 2009; Balaji and Arshinder 2016; Prakash et al. 2017). Some researchers have also used DEMATEL (Sufiyan et al. 2019; Kouhizadeh et al. 2021), while others have applied integrated ISM-DEMATEL (Mangla et al. 2018; Nazam et al. 2020). An integrated ISM and DEMATEL methodology can model multi-criteria problems and categorize the factors into cause-and-effect clusters (Mangla et al. 2018). Other techniques such as dynamic modeling (Gholami-Zanjani et al. 2021a), simulation and modeling (Ivanov 2018), and structural equation modeling (Raut et al. 2021) are also applied in system modeling.

### Research gaps

While the distribution of food commodities and adoption in uncertain environments are critical, FSCs have faced tremendous challenges during the COVID-19 pandemic (FAO 2020). Without affecting operations and providing service, i.e., continuous distribution of safe and secure food to the consumer, the main challenges are the modern food supply chain (FAO 2020; OECD 2020). The modern-day probability of a disruptive event has risen due to complex SCN and globalization (Ivanov et al. 2019). The SC must respond to the disruptive event and quickly restore it to the initial or improved stage (Singh et al. 2020; Gholami-Zanjani et al. 2021a). Demand uncertainty, supplier disruption, and logistics failure are the other issues that affect the food supply chain significantly (Aday and Aday 2020; Richards and Rickard 2020). Many SC failures have been reported in food distribution, quality, and safety to consumers in pandemics (OECD 2020; FAO 2020). Hence, there is a lack of execution of resilience adoption strategy in the FSC. Repurposing SC operations and manufacturing systems is one of the business solutions to deal with pandemic disruption (Poduval et al. 2021). Therefore, identifying key resilient enablers towards preparedness in repurposing the manufacturing system and responding to the pandemic is required (Chowdhury et al. 2021).

The exhaustive literature study indicates that the development of resilience in food SCN is relatively in the nascent stages (Qingbin et al. 2020; Xu et al. 2020a). After determining the importance of enablers for repurposing manufacturing systems through developing resilience, an effort was made to conduct this research to model enablers for resilience building towards pandemic preparedness in FSC. This study combines the Delphi- ISM-MICMAC-fuzzy DEMATEL methodologies to evaluate enablers to develop resilience in FSC. This study contributes to the literature by; a) identifying enablers to develop resilience in the FSC under an uncertain environment in repurposing the manufacturing system and SC operations; b) evaluating and analyzing the contextual interrelationship of the proposed enablers to establish the influential level and hierarchical structure; c) categorizing proposed enablers into the cause-and-effect ones and their influential ranks.

## Research methodology

**Fig. 1:**
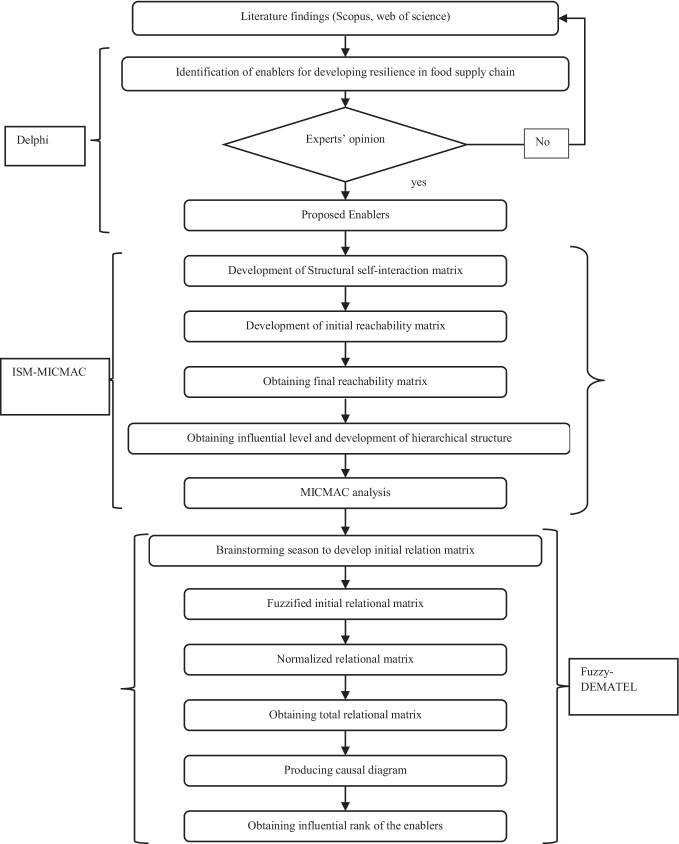
Proposed Research Framework

A hybrid Delphi, ISM, MICMAC, and fuzzy DEMATEL methodology has been utilized in this research. The advantage of the proposed methodology, compared to the other similar techniques, is shown in Table 2. The proposed research framework is shown in Fig. 1*.*

Because of the complexity of the study, we utilized a structured Delphi method to identify the resilience enablers in FSC towards pandemic preparedness. In this study, the following steps were used in the Delphi method.

(i) Identifying enablers from available literature and field survey
(ii) Validating the identified list of enablers through an expert survey. The surveys were repeated until all the experts confirmed the list of enablers. There are no specific rules regarding the number of experts involved in data collection, but Murry and Hammons (1995) suggested ten to 30 while Paul et al. (2021) collected data from ten experts. In this study, we collected data from 60 experts from FSC.
(iii) Obtaining agreement from all experts and finalizing the list of enablers for analysis.

ISM is a systematic method that depends on independent experts to understand the contextual interrelationships among variables (Warfield 1974a). A detailed description of the ISM and DEMATEL methodology is provided in Appendix Sects. 7.1 and 7.2. The fuzzy DEMATEL technique helps reveal the relations between one factor influencing another under uncertain conditions.

The purpose of incorporating the Delphi technique in ISM, MICMAC, and Fuzzy-DEMATEL is to get the best enablers in India's emerging economy (Singh and Sarkar 2020). Delphi technique can sort the best factors by utilizing experts' opinions (Singh and Sarkar 2020). Various researchers have emphasized that ISM and Fuzzy-DEMATEL methods have the edge over other decision-making methods, such as Graph theory, Analytic Network Process (ANP), Analytic Hierarchy Process (AHP), Structural Equation Modeling (SEM), and Interpretive Ranking Process (IRP) (Luthra et al. 2017; Li and Mathiyazhagan 2018; Mangla et al. 2018). The integrated ISM-fuzzy DEMATEL also offers several advantages. First, ISM and DEMATEL can ascertain the relationship between several factors of a complex system. The incorporation of fuzzy helps to deal with uncertainty in decision-making. Second, ISM and DEMATEL can categorize the cause-and-effect variable through ISM's dependence and driving power and relational and prominence matrix in DEMATEL. ISM alone cannot investigate interrelationship deeply; its scale is between 0 and 1, while DEMATEL gives more in-depth information and investigates the strength of contextual interrelationship through a 0 to 4 scale under uncertain or fuzzy environments. The steps to implementing the ISM methodology are given in Sect. 7.1 of Appendix A, while the Fuzzy-DEMATEL methodology is described in Sect. 7.2 of Appendix A.

## Data analysis

### Case study

This study selected the Indian FSC, one of India’s most affected sectors during the COVID-19 pandemic (Singh et al. 2020). Like India, other emerging economies such as Pakistan, Bangladesh, and Nepal have witnessed severe disruption in their food sectors due to the pandemic with increased food prices and insecurities of food commodities. Therefore, this case study is intended for the implication of resilience practice in FSC evident from emerging economic countries towards pandemic preparedness. This research uses expert opinions and an extensive literature review to explore the key enablers useful to develop resilience in FSC. Enablers were also analyzed quantitatively to obtain the most significant enablers and their causal intensities.

### Demographic profile and data collection

Twenty-two enablers for RFSC towards pandemic preparedness were identified from the literature survey. For the Delphi technique, a structured questionnaire was developed for all 22 enablers and sent to experts via e-mail to get their opinion. The questionnaire was based on a 5-point Likert scale. The questionnaire was sent to 75 experts from several food industries and academic institutes. Academic experts with an industrial background and expertise in supply chain management and industry experts with a minimum of graduate qualifications from the food sector were invited. Sixty-four out of 75 experts responded. The study finalized 60 satisfactory responses. Table 3 shows the demographic information of experts. For ISM, data were gathered by a team of six experts (three from academia and three from the food business), who were chosen from a pool of 60 experts to construct a structural self-interaction matrix.

### Delphi study

We identified enablers for developing resilience practice in FSC by getting responses from experts. The responses were analyzed using Microsoft Excel and shown in Table 4. Central tendency parameters, mean, standard deviation, and median were calculated to sort the enablers. Paul et al. (2021) suggested that the threshold of mean and median should not be less than 4 to get accepted from the Delphi study. Therefore, this study takes the same threshold value for selection criteria.

### Resilience enablers identified from Delphi

The 22 enablers were screened through the Delphi study, after which the experts confirmed 14 key enablers as being suitable for the FSC of emerging economies to manage the impacts of the COVID-19 pandemic. The proposed enablers, along with their brief description, are given in Table 5.

### Structural modeling of proposed enablers

#### Step 1: Formation of SSIM

Input for SSIM was obtained from the brainstorming season by a panel of six experts, and one conclusive SSIM was prepared in the form of an upper triangular pairwise comparison matrix. The SSIM matrix is shown in Table 6. In this matrix, relations among two sets of enablers are filled in a symbolic form. SSIM is further converted into IRM, which is shown in Table 12 of Appendix B, followed by the transitivity check to obtain the FRM, which is presented in Table 13 of Appendix B.

#### Step 2: Development of a hierarchical structural model

**Fig. 2:**
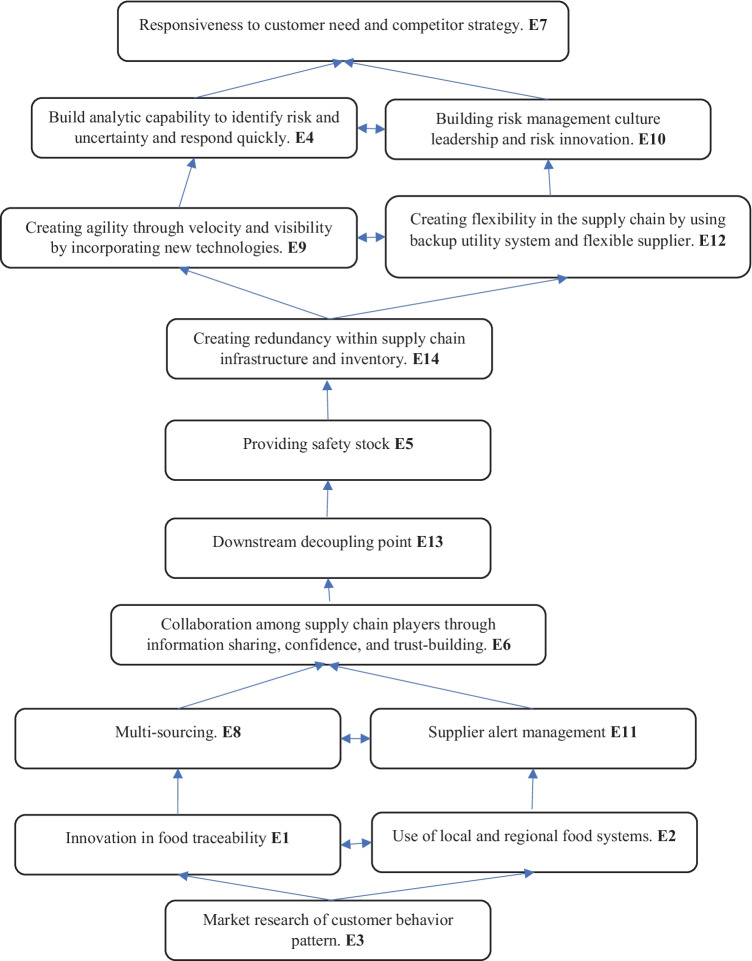
Hierarchical structural model of enablers for FSC resilience

Each enabler's importance level is obtained through level partitioning by matching the reachability set and the interaction set from the FRM, as shown in Table 12 of Appendix B. Enablers were segregated into ten levels, as shown in Table 7. The level of importance of each enabler was grouped into a hierarchy, as shown in Fig. 2. An enabler placed at the bottom of the hierarchy is the most influential, while enablers at the same level have the same importance. The importance of enablers decreases while moving upward in a hierarchical structure.

The hierarchical structure shows that the enabler, ‘market research of customer behavior pattern’ is the most influential enabler, placed at the bottom of the hierarchy. While the two enablers, ‘innovation in food traceability’ and ‘local and regional food system’ are placed at the 9th level, they are critical to developing resilience in the FSC under uncertain environments. The top three enablers are essential in building resilience practice in FSC under uncertain demand conditions by studying customer behavior patterns, using local food systems, and applying traceable systems.

The enablers, ‘multi-sourcing’ and ‘supplier alert management’ were placed at the 8^th^ level and help improve resilience when a disturbance occurs from the supplier side. The enabler ‘collaboration among supply chain players’ provides additional support to build resilience in FSC. Downstream decoupling, providing safety stock, and creating redundancy within supply chain infrastructure and inventory give more rigidity by building resistive capacity and resilience in the face of demand fluctuation. The enablers, ‘creating agility through velocity, viability’ and ‘creating flexibility through flexible supplier and backup utility system’ provide more resilience to FSC. Supply chain operations and manufacturing systems can quickly shift their production lines and processes to produce another high-demand product, thanks to the flexibility provided by flexible suppliers and agility provided by velocity. The extra backup utility system provides greater flexibility to the management when repurposing industrial plants and reduces the financial hurdles to repurposing.

To provide additional force towards pandemic preparedness, enablers such as ‘risk management culture’ and ‘analytical capabilities within the supply chain’ help identify upcoming risks. They encourage quick response to risks, efficiently handle competitors, and meet customer demands. Through strong risk management and good analytical capability, the manufacturing system easily accesses the upcoming risks and opportunities and takes decisive action for repurposing.

#### Step 3: Clustering enablers

**Fig. 3:**
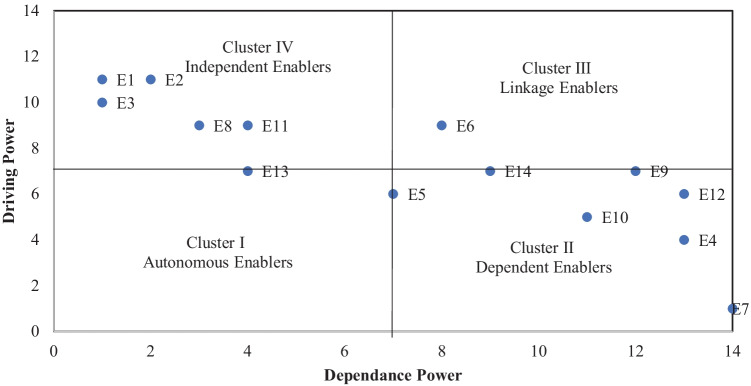
MICMAC analysis

The enablers were clustered into four groups according to the dependence and driving power calculated from the FRM. A matrix cross multiplication analysis and classification- MICMAC analysis was applied to cluster the enablers, as shown in Fig. 3. Enablers with low dependence and driving power were placed in the 1^st^ cluster, known as the autonomous cluster. The enablers found in this cluster are insignificant and may be removed from the study. In our research, no enablers were placed in an autonomous cluster.

The enablers with high dependence and low driving potential belong to the dependent cluster. This study placed enablers E4, E7, E10, and E12 in the dependent cluster. The enablers with high driving and dependence power were placed in the 3^rd^ cluster, known as the linkage cluster. The enablers found in this cluster are highly sensitive to any changes made. Enabler E6 was placed in this cluster. Enablers E14 and E9 were placed at the boundary of the linkage and dependent clusters while E5 is placed at the boundary of autonomous and dependent enablers. The enablers with high driving but low dependence scores were placed in the 4th cluster, i.e., independent cluster. Enablers E1, E2, E3, E8, and E11 were placed in the independent cluster, while enabler E13 was placed at the boundary of the autonomous and independent clusters.

### Classification of the proposed enablers

The MICMAC analysis cannot explore the enablers' causal relationship, and hence, a multi-criteria technique, Fuzzy-DEMATEL, was applied. To develop the initial relational matrix for Fuzzy DEMATEL methodology, a brainstorming session was used to help the panel of experts. After a lengthy discussion on the obtained relationship from FRM, the initial relation matrix in the fuzzy linguistic terms was prepared, as shown in Table 8. After framing the initial relation matrix, it was transformed into a triangular fuzzy number known as the average relation matrix. A normalization technique was used to normalize the fuzzy average matrix denoted by N.

**Fig. 4:**
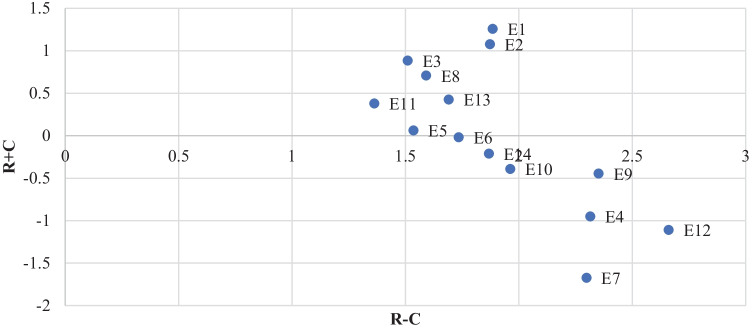
Causal relationship among enablers

A total relational matrix V was obtained using Eq. (3) of Appendix A, given below in Table 9. To obtain the net effect produced (vector R) and net effect conceded (vector C), the row and column values ​​of the respective enablers have been added. The (R + C) and (R—C) vectors were calculated for each prominence and relational vector enabler. A graph between the prominence and relation vector was plotted to classify enablers into cause-and-effect groups, as shown in Fig. 4. The influential rank for all the enablers was calculated using Eq. (5), as given in Appendix A. If the enabler has a positive relational value, it is a part of the cause group, while those with negative relational values are placed in the effect group. The categorization of enablers of RFSC is shown below in Table 10.

From Fig. 4, the enablers E1, E2, E3, E5, E8, E11, and E13 were found to be in the cause group. The enabler, ‘innovation in food traceability,’ had the maximum relational value of 1.258 and was the most influential cause. The enabler, ‘responsiveness to customer need and competitor strategy,’ had the lowest relational (R – C) value of -1.672. According to the prominence (R + C) value, the enablers, ‘creating flexibility within the SC using a backup utility system’ and ‘flexible supplier,’ (2.66) were the most significant ones. In contrast, the enabler, ‘supplier alert management,’ had the lowest prominence value (1.36). The ranking of enablers was obtained by finding the Euclidian distance between two-point (prominence value and relational value) using Eq. (7), as given in Appendix A. The enabler, ‘creating flexibility within the supply chain,’ was placed first, while ‘supplier alert management’ was placed at the end. The ranking of the enablers is shown in Table 10.

## Discussions and implications

This section discusses the findings and managerial implications of the study.

### Discussions on findings

This study employed MICMAC analysis to verify the interrelation hierarchical model obtained from ISM in Fig. 2. It classified the enablers into four groups based on their dependence and driving power. Generally, the independent enablers are placed at the bottom of the hierarchy that actuates the system. Seven enablers, i.e., ‘innovation in food traceability,’ ‘local and regional food use,’ ‘market research on customer behavior pattern,’ ‘providing safety stock,’ ‘multi-sourcing,’ ‘supplier alert management,’ and ‘downstream decoupling point,’ were selected as ‘cause enablers’ with positive relational values. They should be prioritized while developing resilience in FSCs. The MICMAC analysis revealed six enablers, i.e., ‘innovation in food traceability,’ ‘use of local and regional food,’ ‘market research on customer behavior pattern,’ ‘multi-sourcing,’ ‘supplier alert management,’ and ‘downstream decoupling point,’ to be in the independent cluster. On the other hand, the enabler, ‘providing safety stock,’ was placed at the boundary of the autonomous and dependent cluster. The analysis is in line with earlier findings that emphasized that the causal factors should be identified in the independent cluster in MICMAC (Mangla et al. 2018). In the structural model developed through ISM in Fig. 2, the enablers placed at the bottom are largely independent and causal factors. Hence, FSC needs to focus more on these causal factors in developing resilience to respond better to global disruptions like pandemics (Raut et al. 2021).

Innovation in food traceability and adoption is of the utmost importance since it ensures authenticity, privacy, and reliability, which help in building resilience in an uncertain environment. Technologies like AI and blockchain act as enablers to improve traceability and support preparedness during pandemics Naz et al. (2021). Traceability is a foundational step for underpinning operational safety. Traceability helps enhance FSC resilience by documenting immutable and tamper-proof information related to food safety, fraud, loss, and wastage. ‘Innovation in food traceability’ is essential as it provides real-time tracking in the supply chain network (SCN), thus helping to maintain the quality and safety of food products (Saberi et al. 2019). The use of local and regional food systems can help respond adequately in response to the fragility of FSC (Bene 2020). Disruptive events like droughts and floods adversely affect the operating efficiency of the various actors (food producers, retailers, transporters) in local and regional FSC. In such scenarios, collaboration with local food suppliers helps mitigate logistics and supply risks (Hobbs 2020; Dirsehan and Cankat 2021). ‘Market research on customer behavior patterns’ is critical since it helps understand customer demand patterns, thereby establishing stronger customer bonds (Gölgeci and Kuivalainen 2020). In a pandemic-like situation, the consumer behavior pattern changes, and the enabler, ‘market research on customer behavior patterns,’ is key to pandemic preparedness and building resilience in FSC. Also, the demand for essential products increases during a pandemic. Hence, there is a need to rethink production planning and the manufacture of different products (Throup et al. 2021). It is also evident that with advancements in technology and better market research, firms can share timely information with both suppliers and consumers and help enhance SC performance (Brandon-Jones et al. 2014). The fluctuations in demand and supply between suppliers, retailers, and consumers, especially during mass disruptions, can be answered if firms can switch suppliers to fulfill consumer demands (Baihaqi and Sohal 2013). Providing safety stock helps reduce uncertainty by checking supply chain volatility, thereby guaranteeing consumer safety and satisfaction.

Multi-sourcing allows supply chains to recuperate, enhance their resilience level, and provide strength, while battling a huge disruption like the COVID-19 pandemic. The multi-sourcing failure of one node of the SC did not affect the whole SCN. The multi-sourcing enablers play a massive role in repurposing the manufacturing system, providing flexibility and resilience to the FSC (Mahroof et al. 2021). Supplier alert management supports integration between suppliers and other stakeholders. The digital or AI-enabled supplier alert management ensures timely information sharing and alert management, which helps respond efficiently and take appropriate actions, thus yielding a resilient supply chain (Kim and Chai 2017). During a pandemic, AI-driven digital supplier alert management proves a resilient system and helps in preparedness and repurposing.

Downstream decoupling point helps to optimally site multiple storages and warehouse locations to suit different stakeholders, and ensures better market penetration. In unexpected events and mass disruptions, emergency/ backup supplies that are stored at the other facility can be transported to locations that need to be served more rigorously (Hale and Moberg 2005).

Seven enablers, namely, ‘building analytic capability to identify risk and uncertainty and respond quickly,’ ‘collaboration among supply chain players through information sharing, confidence, and trust-building,’ ‘responsiveness to customer need and competitor strategy,’ ‘creating agility through velocity and visibility by incorporating new technologies,’ ‘building risk management culture leadership and risk innovation,’ ‘creating flexibility within supply chain by using backup utility system and flexible supplier,’ and ‘creating redundancy within supply chain infrastructure and inventory,’ were found to be in the effect category because of negative (R–C) values. Therefore, in MICMAC analysis, enablers E4, E10, and E12 were in the dependent cluster, while E9 and E14 were found at the boundary of the dependent and linkage clusters. ‘Collaboration among supply chain players through information sharing, confidence, and trust-building’ was the only enabler found in the linkage cluster. Moreover, ‘collaboration and information sharing’ help firms reboot after a pandemic and build resilience capacity. Through the SC collaboration and information-sharing, barriers of repurposing manufacturing systems, such as worker unavailability, sharing of knowledge, and unavailability of skilled workers, can be overcome (Poduval et al. 2021). In ISM structural hierarchy, mostly independent enablers are placed at the top of the hierarchy, depending on the lower level of enablers. ‘Responsiveness to customer needs and competitor strategy’ was found at the top of the hierarchy. ‘Build analytic capability to identify risk and uncertainty quickly,’ and ‘building risk management culture leadership and risk innovation’ were at the second level of hierarchy. To develop resilience within the FSC, these effect enablers should be minimized by improving their cause. The enablers, ‘building digital risk management’ and ‘building analytical capability,’ help manufacturing systems mitigate technological barriers for repurposing (Poduval et al. 2021).

Multi-sourcing can be improved by implementing innovative technologies like blockchain and IoT-driven systems, using local suppliers that can improve customer responsiveness, create agility, and increase flexibility within FSC (Singh et al. 2020). The multi-sourcing enablers help overcome resource constraints such as manpower, material, and supply chain unavailability, allowing the production operation to simplify its repurposing strategy (Poduval et al. 2021). With increased manufacturing system flexibility, it can repurpose easily by switching production processes and layout to create new high-demand products. In addition, the flexible manufacturing system makes it easier to transfer from one production process to another during a pandemic. This research's outcomes, such as contextual relationship, cause-effect categorization, and the hierarchical model, have been discussed with various experts in FSC and found to be in line with their understanding.

### Implications for theory

This study has various implications for the theoretical point of view in FSC, while building resilience, pandemic preparedness, and repurposing the manufacturing system. The enabler, ‘market research of customer behavior,’ is critical in FSC during a pandemic as it helps implement food traceability for transparency, safety, and quality. Traceability is an integrated part of the logistic system and acts as a causal enabler in developing resilience in the FSC (Bosona and Gebresenbet 2013; Saurabh and Dey 2020; Kouhizadeh et al. 2021). Also, integrating local and regional food systems into the FSC helps develop resilience. This is analogous to Thilmany et al*.* (2021). Local and regional food systems help and enable FSC to use multiple-sourcing that help in planning for preparedness as well as repurposing. Several researchers (Ali et al. 2017; Singh et al. 2018, 2020; Taleizadeh et al. 2021) have discussed that multi-sourcing is the best approach during supplier side disruption, functioning as suppliers’ backup option. Also, multi-sourcing helps to source different raw materials from different suppliers as required when repurposing the production process. It is also worth noting that if one supplier fails due to any reason, the regional supplier can be synced for uninterrupted supplies.

### Implications for practitioners and policy recommendation

This research's outcomes are significant for the development of resilience practices in the Indian FSC under uncertain environments, especially the present pandemic. This work helps answer the research questions, i.e., identifying enablers, finding hierarchies, and examining causal relationships among them. The research has followed a sequential approach to investigate and authenticate the information obtained from literature and expert judgments. The previous research designs have predominantly focused on cross-sectional and qualitative methods, while the present work developed a unique methodology. Future works can employ this methodology to explore the different enablers influencing FSC comprehensively. The Delphi-ISM-Fuzzy-DEMATEL model is distinctive and has a multifold contribution to the literature.

- The enablers placed in the independent cluster were strategy-oriented, while enablers placed in the dependent cluster were performance-orientated. Five enablers, including ‘innovation in food traceability, ‘use of local and regional food system,’ ‘market research of customer behavior pattern,’ ‘multi-sourcing,’ and ‘supplier alert management,’ were kept in a separate category. They are strategically oriented and help build strategy while preparing for a pandemic or repurposing their manufacturing operations. Three enablers, ‘build analytic capability to identify risk and uncertainty and respond quickly, ‘building risk management culture,’ and ‘downstream decoupling point,’ were found to be performance-oriented. Therefore, risk identification, risk analysis, risk evaluation, and real-time alert management can help decision-makers take action based on the criticality of the risk through building analytical capability in the food system using cloud computing, data analytics, and AI. Similarly, the downstream decoupling point helps buffer stock for the FSC and supports demand uncertainty.
- The findings of this study reveal how managers may use resilience practice to modify their manufacturing operations. Repurposing strategy can be implemented quickly and accurately by keeping the production system adaptable, adding a backup utility system, working with a flexible supplier, encouraging coordination among partners, having a risk management culture, and strong analytical capabilities.
- The solution focuses on the enablers of critical importance, i.e., ‘market research on customer behavior pattern,’ ‘innovation in food traceability,’ and ‘use of local and regional food systems.’ This will assist in building resilient FSC with an uninterrupted food supply to fulfill human needs, even during mass disruptions. The above-discussed enablers are critical in repurposing and preparedness for a pandemic. The food system should focus on digitalization to improve traceability and velocity to provide the best service and satisfaction. Food systems may create redundancy in supply chain infrastructure, and inventory helps during uncertainty in supply and demand, thereby increasing resilience.
- Further, the enablers were ranked and categorized into cause-effect groups. The research suggests that a resilient FSC can be built by focusing on the enablers of critical importance.

#### Policy recommendations

Based on the research findings, several policies have been recommended for the Indian FSC for pandemic preparedness and repurposing. Food industries should prioritize research in their system, especially research on customer behavior, to understand choice and satisfaction level. The customer behavior study provides manufacturers with the leverage to provide appropriate taste and quality of food and increase customer satisfaction. The food supply chain requires transparency by implementing traceability with advanced technologies like IoT, blockchain, and artificial intelligence. The advanced level of transparency, collaboration, and trust among the stakeholders of the FSC may improve. The collaboration and trust among the supply chain stakeholders provide strength and support during pandemics in terms of finance, technology, etc. Based on the study's findings, food manufacturing firms are advised to source raw material from multiple suppliers and use local and regional food systems where possible. Multi-sourcing is very helpful in the supplier disruption periods or when planning to repurpose the manufacturing system. National and international borders get closed when the local or regional food supplier supplies in the pandemic. The dairy industry is a successful example of local and regional food suppliers. They were not as affected as other industries during the pandemic period.

It is recommended that food manufacturing firms implement intelligent supplier alert management systems with advanced industry 4.0 technology. Food manufacturing firms are also advised to keep safety stock and downstream decoupling points. The decoupling point in the downstream supply chain refers to the multiple storage facilities and control systems between the manufacturer and the market. Food manufacturing firms should use data analytic capability through AI, cloud computing, and big data that help in building intelligent risk management. Intelligent risk management can quickly identify, classify, and alert on time, thus easing the planning process.

## Conclusions and directions for future research

It is important to investigate how a disaster-resilient system can be developed to survive unexpected events such as the recent pandemic. The growing concern over disruptions, especially in the FSC, needs to fuel the development of initiatives to overcome such situations. In this pandemic situation, manufacturing operations fight for their existence in the market; therefore, restructuring and repurposing their operations can help them survive during a crisis. Hence, this study investigates critical enablers for developing resilience initiatives in the FSC for repurposing and preparedness for the pandemic or even multiple transitions. Twenty-two enablers were selected primarily through literature and sorted into 14 through responses taken from several experts from the FSC academia and food industry practitioners. Identifying and analyzing the critical resilient enablers for FSC to aid pandemic preparedness makes this research unique. Also, through the DEMATEL methodology, the causal interaction along with their intensity value was analyzed. The enablers were analyzed using the integrated ISM–fuzzy DEMATEL approach. ISM methodology was applied to identify the contextual interrelationships and develop the proposed enablers' hierarchical structure. The ISM's findings imply that the enabler, ‘market research of the customer behavior pattern’ is the most significant enabler and is placed at the bottom of the hierarchy, while ‘responsiveness to customer need and competitor strategy.’ is placed bottom the top level of the least influential hierarchy.

Further, the Fuzzy-DEMATEL methodology was applied to other grouping enablers to reveal the cause and effect interrelationships among the proposed enablers. This methodology showed that seven enablers were segregated in the cause category. The enabler, ‘innovation in food traceability,’ had the highest relational score (R—C, 1.258), while ‘providing safety stock’ had the most negligible R – C value (0.062). Seven enablers were placed in the effect category. The enabler, ‘responsiveness to customer need and competitor strategy,’ had the least relational score (R – C, -1.67), while ‘collaboration among supply chain players through information sharing, confidence, and trust-building’ had the highest negative relational score (R – C is -0.019). Therefore, it is recommended that food systems research customer relationship management to read consumers' behavior. They should also look into FSC digital technologies-based analytic capability for real-time risk evaluation and decision making.

The proposed research framework may help in building resilience practice in FSC to ensure pandemic preparedness. This paper provides insights into understanding the enablers’ hierarchical structure, importance level, and contextual relationship. The cause-effect grouping and interrelationship may vary with a different set of experts because of subjectivity. Fuzzy theory can be employed on MICMAC to yield a different result.

## Appendix A

### Interpretive structural modelling (ISM)

ISM was first recommended by Warfield (1974b) and used for modeling the relationship between several factors in a complex system that is unstructured and poorly communicated. Several researchers previously applied ISM in different fields of supply chain risk assessment (Prakash et al. 2017), causal factors of food losses (Balaji and Arshinder 2016; Gardas et al. 2018) to model success factors in various areas (Gardas et al. 2019; Gopal and Thakkar 2016; Luthra et al. 2018); and in modeling enablers (Mangla et al. 2018). The ISM has the following steps that are-

(i) Structural self-interaction matrix’s (SSIM) development
  The SSIM is established to obtain the relationship between two enablers by filling the pairwise comparison matrix's upper-triangular cell. A brainstorming session with a team of 6 experts helped fill the conclusive SSIM matrix. Alphabetical characters (such as V, A, X, and O) are utilized to fill the SSIM matrix. All alphabetical characters are used by their significance which are: when enabler 'p' helps to achieve enablers ‘q’ V becomes the cell value when enabler 'q' help to achieve enabler 'p' the cell value is A, if both the enablers help each other then character 'X' is used while if both the enablers are unrelated or neither helps the other, the character ‘O’ is used to fill the cell (i, j).
(ii) Obtaining initial reachability matrix- IRM
  The Initially developed SSIM has cell values filled with alphabetical symbols converted into binary digits (0 & 1). The obtained binary matrix is the initial reachability matrix- IRM. The transformation of alphabetical symbols is obtained by using some set of rules which are:
  If the cell value (p, q) has symbol ‘V’, then cell (p, q) is filled with 1 and cell (q, p) is filled with 0
  If the cell value (p, q) has symbol ‘A’, then cell (p, q) is filled with 0 and cell (q, p) filled with 1
  If the cell value (p, q) has symbol ‘X’, then cell (p, q) is filled with 1 and cell (q, p) filled with 1
  If the cell value (p, q) has symbol ‘O’, then cell (p, q) is filled with 0 and cell (q, p) filled with 0
(iii) Formation of final reachability matrix (FRM).
  A transitivity check is performed on IRM to verify the relationship among the enablers. A transitivity check was performed manually by checking all the cell values with an entry of 0, which means no relation. Transitivity check is performed in such a way that if enabler ‘e1’ is related to enabler ‘e2’ and ‘e2’ is related to enabler ‘e3’ then enabler ‘e1’ must be in relation with ‘e3,’ in which case the cell must be filled with 1* by summing individual column and row entry values to get dependence and driving power of enabler.
(iv) Level partitioning and development of hierarchical structure
  The antecedent and reachability set of the enablers was matched to obtain the intersection set. If the intersection set value of such enablers is equal or similar to the reachability set, it is named the enablers' first level. The same iteration is performed by removing the enablers that are already leveled and the next level of enablers is found till all the enablers are not leveled.
  The hierarchy is formed by keeping the first level of enablers at the top. One level of enablers is linked with an arrow in the upward direction, while enablers at the same level are linked with the bi-relational arrow.
(v) MICMAC analysis
  Matrix cross multiplication and classification- MICMAC analysis is performed to cluster the enablers in 4 different groups as per their dependence (DEP) and driving power (DRIV) scores. By plotting these power scores, the final reachability matrix and MICMAC graph are obtained. Graphs are divided into four quadrants, namely 1^st^ Autonomous cluster, second Dependent cluster, third Linkage cluster, and Independent cluster. Enablers having lower DEP and DRIV power are placed in autonomous clusters and enablers who get high DEP and low DRIV power are generally put at dependent clusters. Enablers with high DRIV and DEP values are placed at linkage clusters while those with high driving power but low dependence power are placed at independent clusters.

### DEMATEL

DEMATEL methodology is utilized to explore the causal relationship of a complex problem. DEMATEL helps to compute relationships and strengths among the factors. DEMATEL methodology also categorizes the factors into two groups and develops a causal relationship model (Gardas et al. 2018). In this research, fuzzy logic is used with DEMATEL to overcome imprecision in decision-making. Following are the steps in DEMATEL:

(i) Initial relational matrix’s development
  IRM is generated by building a pairwise comparison matrix on the five-point fuzzy scale between 0–4 (where ‘0’ – ‘no influence’ & ‘4’ – ‘very high influence’). A five-point linguistic scale is given in Table 11. A triangular fuzzy number (l, m, u) or (lower, medium, upper) is utilized. The initial relational matrix is formed through the judgment of the panel of several experts.

**Table 11:** Triangular fuzzy number

Linguistic value	Notation	Triangular fuzzy number
No effect	0	(0,0,0.25)
Low effect	1	(0,0.25,0.5)
Medium effect	2	(0.25,0.5,0.75)
High effect	3	(0.5,0.75,1)
Very high effect	4	(0.75,1,1)

(ii) Obtaining average relation matrix
  The average relationship matrix ‘G’ is obtained after getting the inputs from all the experts using Eq. (1). Let there be an H number of experts so there is an h pairwise comparison matrix. First, the entire matrix is converted into a triangular fuzzy number. Let (x^h^_ij_) be the cell value of i^th^ row and j^th^ column of the h^th^ expert where h belongs to $1\le h\le H$. Then the average relation matrix G can be established as Eq. (1).
  1 $$G=\frac{\sum_{h=1}^{H}{x}_{ij}^{h}}{H}$$
(iii) Obtaining normalized relation matrix NRM
  The NRM, ‘N’ can be obtained using Eq. (2)
  2 $${\mathrm{N}}_{\mathrm{ij}}=\frac{{\mathrm{g}}_{\mathrm{ij}}}{\mathrm{r}}$$
  where
  $\mathrm{r}={\mathrm{max}}_{1\le \mathrm{i}\le \mathrm{n}}\sum_{\mathrm{j}=1}^{\mathrm{n}}{\mathrm{u}}_{\mathrm{ij}}$ and ${\mathrm{u}}_{\mathrm{ij}}$ is the upper value triangular fuzzy number of i^th^ row and j^th^ column.
(iv) TRM Development
  Total relation matrix ‘V’ can be developed using Eq. (3)
  3 $$\mathrm{V}={\mathrm{N}(\mathrm{I}-\mathrm{N})}^{-1}$$
  where ‘I’ is the unit matrix.
(v) Obtaining prominence, relational vector, and rank of enablers.
  For obtaining prominence and relational vector, the summation of columns (c) and rows (r) of TRM is obtained by using Eqs. (4) and (5)
  4 $$R={\left[{\textstyle\sum_{j=1}^I}\;t_{ij}\right]}_{n\times1}$$
  5 $$C={\left[{\textstyle\sum_{j=1}^I}\;t_{ij}\right]}_{1\times{n}}$$
  Defuzzification of fuzzy numbers into crisp values is performed through Eq. (6).
  6 $${x}_{Def}=\left(l+\frac{\left(m-l\right)+(u-l)}{3}\right)$$
  R denotes the total effect provided by the enabler (p) to (q), and C denotes the total effect gained by enablers (q) from (p). By summation of R and C, (R + C) prominence vector is obtained; the difference between R and C, (R–C) gives the relation vector. The rank of enablers is calculated by prioritizing the influence score obtained from Eq. (7).
  7 $$Influence\; score=\sqrt{{(R+C)}^{2}+{(R-C)}^{2}}$$
(vi) Producing causal diagram
  The causal diagram is produced by plotting a graph between prominence value and relation vector. Relation vector is used to categorize enablers into cause and effect. When the value of the relational vector for an enabler is positive, it is grouped as a causal enabler, and if it is negative, it is grouped among effect enablers.

## Appendix B

### Step- 1 Obtaining IRM for enablers used in the development of resilience in FSC

The initial reachability matrix was obtained by transforming alphabetical symbols used in SSIM to binary digits. Conversion of SSIM into IRM follows the rule discussed above in Sect. 7.1 point ii. The converted IRM is shown in Table 12.

**Table 12:** IRM for the enabler used in the development of resilience in the FSC

	E14	E13	E12	E11	E10	E9	E8	E7	E6	E5	E4	E3	E2	E1
E1	1	1	1	1	0	1	0	1	1	0	1	0	0	1
E2	1	0	1	1	0	1	1	1	0	0	1	0	1	0
E3	1	0	0	0	0	0	0	1	0	0	1	1	1	0
E4	0	0	0	0	1	0	0	1	0	0	1	0	0	0
E5	1	0	1	0	0	0	0	1	0	1	0	0	0	0
E6	0	1	1	0	1	0	0	1	1	0	1	0	0	0
E7	0	0	0	0	0	0	0	1	0	0	0	0	0	0
E8	1	0	1	0	0	1	1	1	0	1	0	0	0	0
E9	0	0	0	0	1	1	0	0	1	0	1	0	0	0
E10	0	0	1	0	1	0	0	0	0	0	1	0	0	0
E11	0	0	1	1	1	1	0	1	0	1	1	0	0	0
E12	0	0	1	0	0	1	0	1	0	0	1	0	0	0
E13	1	1	1	0	0	0	0	1	0	1	0	0	0	0
E14	1	0	1	0	0	1	0	1	0	0	1	0	0	0

### Step- 2 Obtaining FRM

A FRM is obtained by performing a transitivity check. A transitivity check is performed for all the IRM cells whose entry value is 0 (not in relation); the procedure has been discussed in Sect. 7.1, point iii. FRM for enablers for the development of resilience in FSC is shown in Table 13.

**Table 13:** Final reachability matrix for the enablers of resilience in the food supply chain

	E1	E2	E3	E4	E5	E6	E7	E8	E9	E10	E11	E12	E13	E14
E1	1	0	0	1	1*	1	1	0	1	1*	1	1	1	1
E2	0	1	0	1	1*	1*	1	1	1	1*	1	1	0	1
E3	0	1	1	1	0	0	1	1*	1*	1*	1*	1*	0	1
E4	0	0	0	1	0	0	1	0	0	1	0	1*	0	0
E5	0	0	0	1*	1	0	1	0	1*	0	0	1	0	1
E6	0	0	0	1	1*	1	1	0	1*	1	0	1	1	1*
E7	0	0	0	0	0	0	1	0	0	0	0	0	0	0
E8	0	0	0	1*	1	1*	1	1	1	1*	0	1	0	1
E9	0	0	0	1	0	1	1*	0	1	1	0	1*	1*	0
E10	0	0	0	1	0	0	1*	0	1*	1	0	1	0	0
E11	0	0	0	1	1	1*	1	0	1	1	1	1	0	1*
E12	0	0	0	1	0	1*	1	0	1	1*	0	1	0	0
E13	0	0	0	1*	1	0	1	0	1*	0	0	1	1	1
E14	0	0	0	1	0	1*	1	0	1	1*	0	1	0	1

1* is the new entry after transitive check

## Appendix C

**Table 14:** Past Studies on Modeling Enablers in supply chain

Author	Factor type	Tools and technique	Area of study	Objective
Magalhães et al. (2021)	Cause of food waste	ISM	Fruit & vegetable	Modeling the cause of food wastage in fruit and vegetable SC
Majumdar et al. (2021)	Risk Factors	Analytic Hierarchy Process (AHP)	Clothing supply chains	Modelling risk factors in green clothing supply chain
Parashar et al. (2020)	Enablers	ISM-MICMAC	Food supply chain	Modeling of enablers for reducing the carbon footprint
Yang and Lin (2020)	Drivers	ISM-MICMAC	automobile firm	Analyzing drivers of green innovation practice
Singh et al. (2020)	Enablers	Fuzzy Delphi-DEMATEL	Automobile firm	Sustainable product development
Gardas et al. (2019)	CSF	TISM-MICMAC	Reusable plastic packaging	Identifying CSF for obtaining reusable plastic packaging in sustainable SC
Raj and Sah (2019)	CSF	Gray DEMATEL	Field Study	CSF for implementation of drone in the logistic system
Raut et al. (2019)	KPI	Confirmatory factor analysis	Agri-Food (Fruit)	Identifying the performance inhabitors in Indian Agri green management practice
Mangla et al. (2018)	Enablers	ISM-Fuzzy-DEMATEL	Agri-Food (Fruit)	Modeling enablers for sustainable development iterative
Shankar et al. (2018)	CSF	Exploratory factor analysis and TISM	Food supply chain	Modeling CSF for traceability implementation in the logistic system
Grimm et al. (2018)	CSF	DEMATEL	Field Study	Establishing relationship among CSF of sub-supplier corporate sustainability standards
Gardas et al. (2018)	Cause of Food waste	DEMATEL	Agri-food	Modeling Post-harvest loss drivers in Indian agri-food
Raut et al. (2017)	CSF	ISM-MICMAC	Oil and gas industry	Modeling CSF in the oil and gas industry for sustainable development practice
Yadav and Barve (2015)	CSF	ISM-MICMAC	Humanitarian SC	Modeling CSF in humanitarian SC in disaster preparedness
